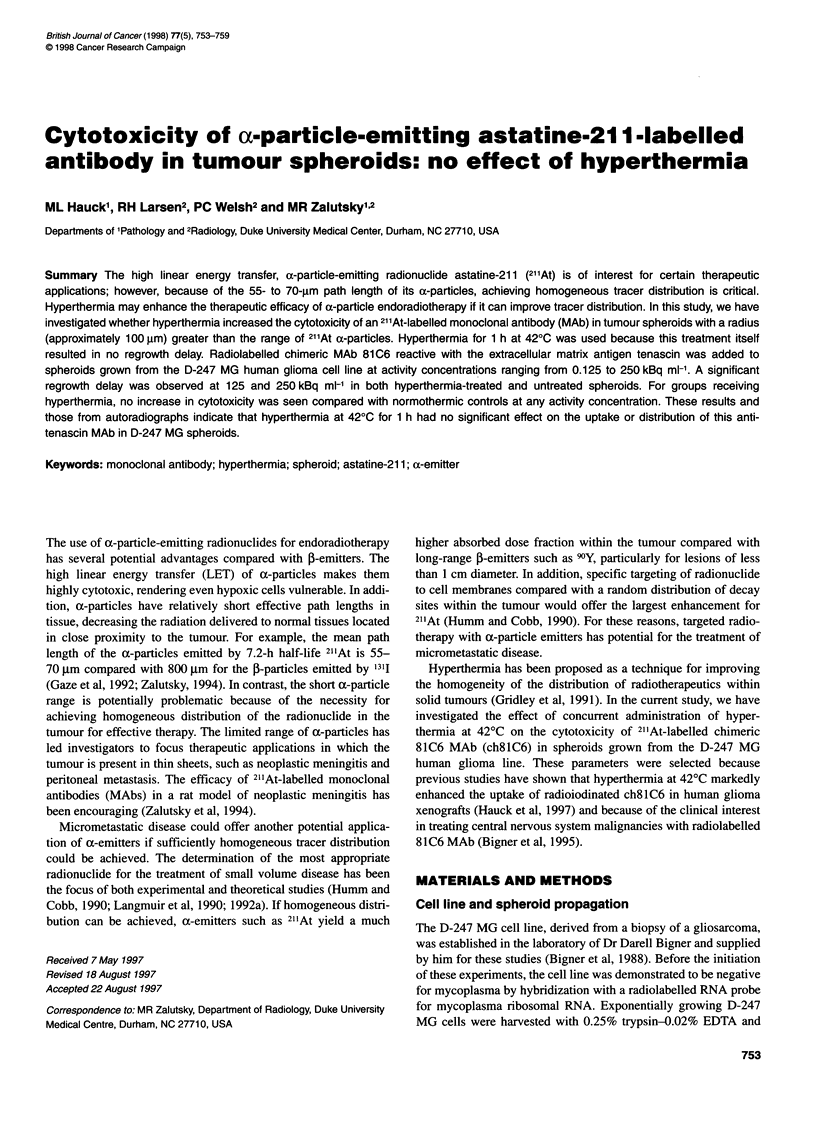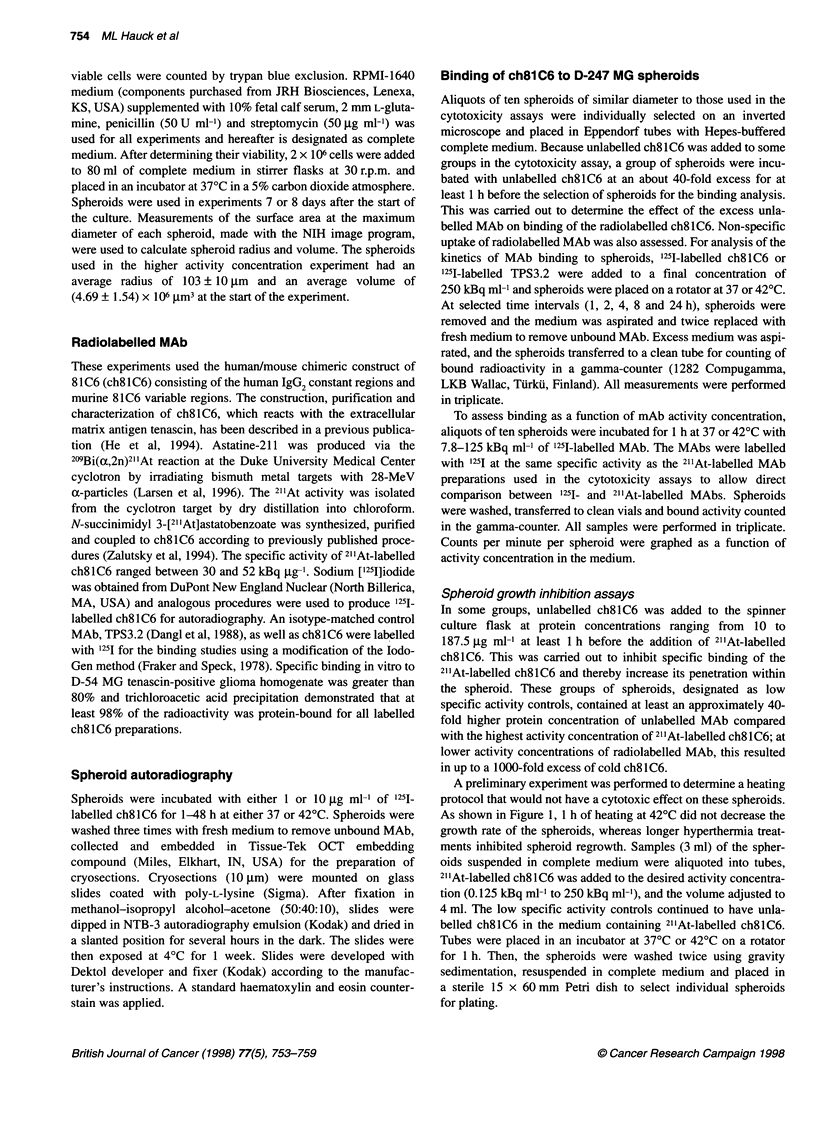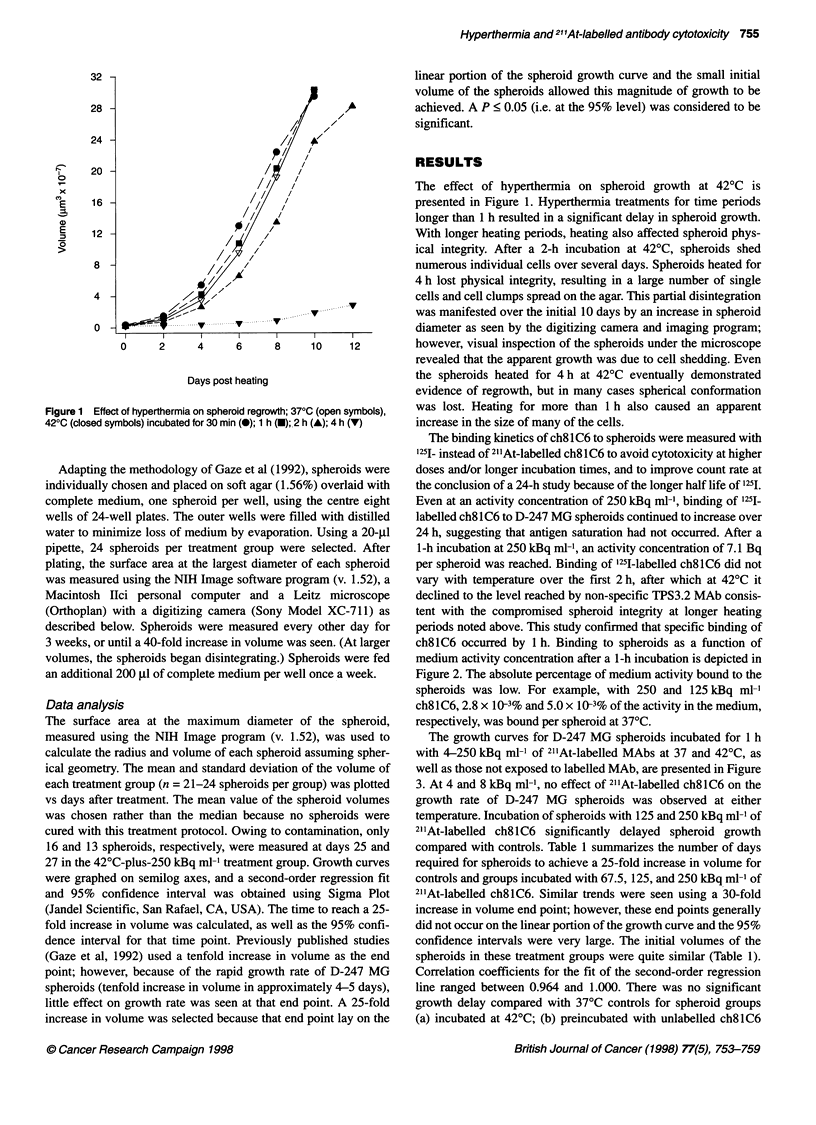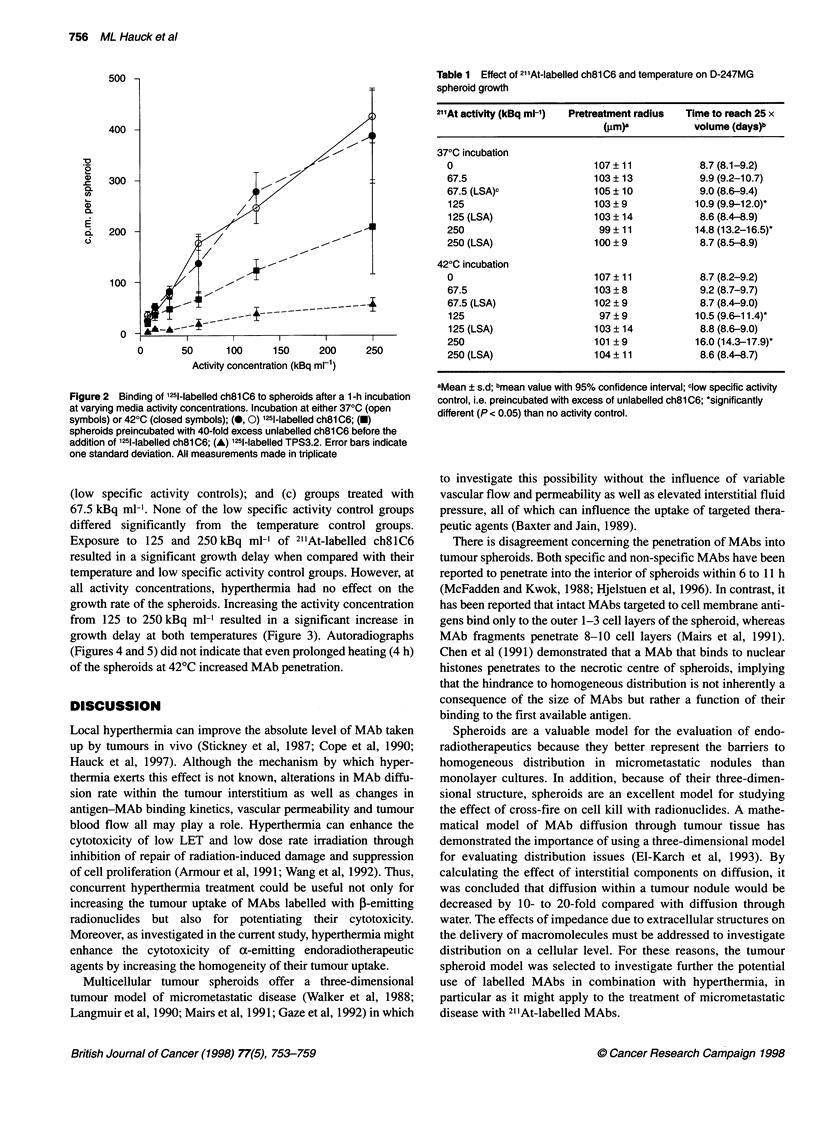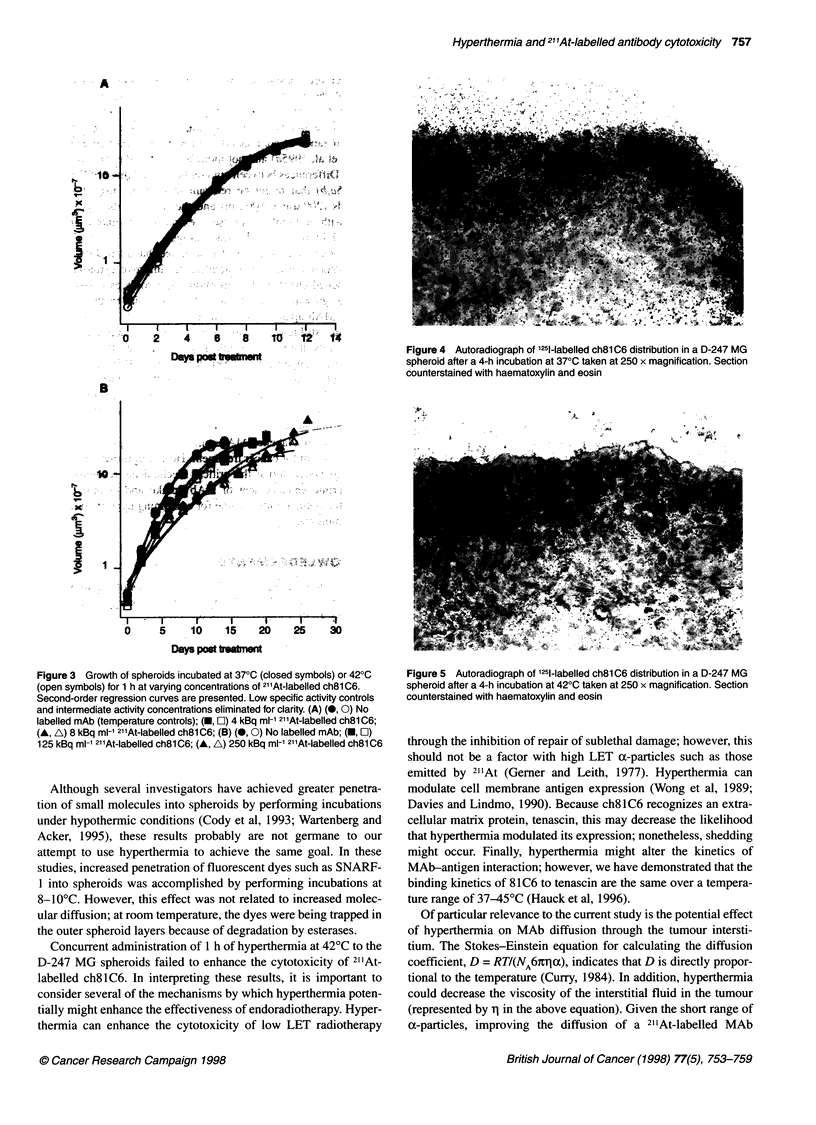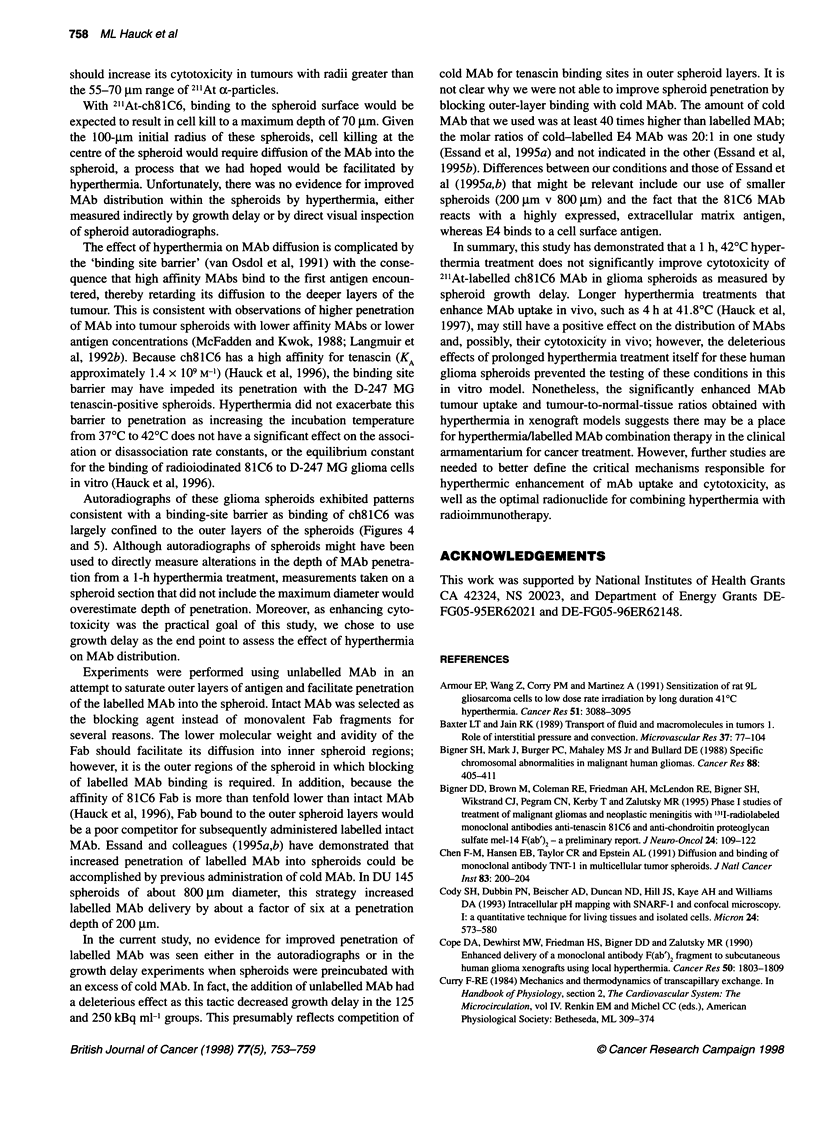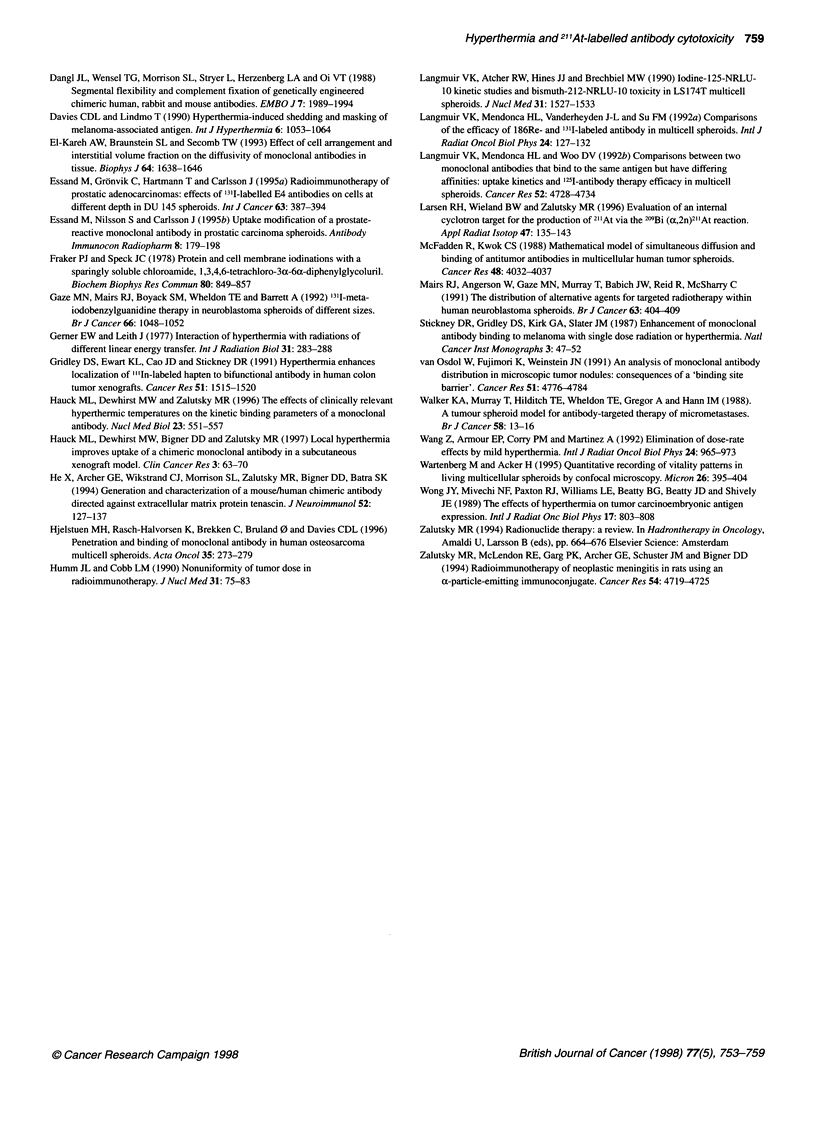# Cytotoxicity of alpha-particle-emitting astatine-211-labelled antibody in tumour spheroids: no effect of hyperthermia.

**DOI:** 10.1038/bjc.1998.123

**Published:** 1998-03

**Authors:** M. L. Hauck, R. H. Larsen, P. C. Welsh, M. R. Zalutsky

**Affiliations:** Department of Pathology, Duke University Medical Center, Durham, NC 27710, USA.

## Abstract

**Images:**


British Journal of Cancer (1998) 77(5), 753–759
© 1998 Cancer Research Campaign

### Cytotoxicity of α-particle-emitting astatine-211-labelled antibody in tumour spheroids: no effect of hyperthermia

**ML Hauck[1], RH Larsen[2], PC Welsh[2] and MR Zalutsky[1,2]**

Departments of [1]Pathology and [2]Radiology, Duke University Medical Center, Durham, NC 27710, USA

**Summary** The high linear energy transfer, α-particle-emitting radionuclide astatine-211 ($^{211}At$) is of interest for certain therapeutic applications; however, because of the 55- to 70-µm path length of its α-particles, achieving homogeneous tracer distribution is critical. Hyperthermia may enhance the therapeutic efficacy of α-particle endoradiotherapy if it can improve tracer distribution. In this study, we have investigated whether hyperthermia increased the cytotoxicity of an $^{211}At$-labelled monoclonal antibody (MAb) in tumour spheroids with a radius (approximately 100 µm) greater than the range of $^{211}At$ α-particles. Hyperthermia for 1 h at 42°C was used because this treatment itself resulted in no regrowth delay. Radiolabelled chimeric MAb 81C6 reactive with the extracellular matrix antigen tenascin was added to spheroids grown from the D-247 MG human glioma cell line at activity concentrations ranging from 0.125 to 250 kBq $ml^{-1}$. A significant regrowth delay was observed at 125 and 250 kBq $ml^{-1}$ in both hyperthermia-treated and untreated spheroids. For groups receiving hyperthermia, no increase in cytotoxicity was seen compared with normothermic controls at any activity concentration. These results and those from autoradiographs indicate that hyperthermia at 42°C for 1 h had no significant effect on the uptake or distribution of this anti-tenascin MAb in D-247 MG spheroids.

**Keywords:** monoclonal antibody; hyperthermia; spheroid; astatine-211; α-emitter

The use of α-particle-emitting radionuclides for endoradiotherapy has several potential advantages compared with β-emitters. The high linear energy transfer (LET) of α-particles makes them highly cytotoxic, rendering even hypoxic cells vulnerable. In addition, α-particles have relatively short effective path lengths in tissue, decreasing the radiation delivered to normal tissues located in close proximity to the tumour. For example, the mean path length of the α-particles emitted by 7.2-h half-life $^{211}At$ is 55–70 µm compared with 800 µm for the β-particles emitted by $^{131}I$ (Gaze et al, 1992; Zalutsky, 1994). In contrast, the short α-particle range is potentially problematic because of the necessity for achieving homogeneous distribution of the radionuclide in the tumour for effective therapy. The limited range of α-particles has led investigators to focus therapeutic applications in which the tumour is present in thin sheets, such as neoplastic meningitis and peritoneal metastasis. The efficacy of $^{211}At$-labelled monoclonal antibodies (MAbs) in a rat model of neoplastic meningitis has been encouraging (Zalutsky et al, 1994).

Micrometastatic disease could offer another potential application of α-emitters if sufficiently homogeneous tracer distribution could be achieved. The determination of the most appropriate radionuclide for the treatment of small volume disease has been the focus of both experimental and theoretical studies (Humm and Cobb, 1990; Langmuir et al, 1990; 1992a). If homogeneous distribution can be achieved, α-emitters such as $^{211}At$ yield a much higher absorbed dose fraction within the tumour compared with long-range β-emitters such as $^{90}Y$, particularly for lesions of less than 1 cm diameter. In addition, specific targeting of radionuclide to cell membranes compared with a random distribution of decay sites within the tumour would offer the largest enhancement for $^{211}At$ (Humm and Cobb, 1990). For these reasons, targeted radiotherapy with α-particle emitters has potential for the treatment of micrometastatic disease.

Hyperthermia has been proposed as a technique for improving the homogeneity of the distribution of radiotherapeutics within solid tumours (Gridley et al, 1991). In the current study, we have investigated the effect of concurrent administration of hyperthermia at 42°C on the cytotoxicity of $^{211}At$-labelled chimeric 81C6 MAb (ch81C6) in spheroids grown from the D-247 MG human glioma line. These parameters were selected because previous studies have shown that hyperthermia at 42°C markedly enhanced the uptake of radioiodinated ch81C6 in human glioma xenografts (Hauck et al, 1997) and because of the clinical interest in treating central nervous system malignancies with radiolabelled 81C6 MAb (Bigner et al, 1995).

### MATERIALS AND METHODS

### Cell line and spheroid propagation

The D-247 MG cell line, derived from a biopsy of a gliosarcoma, was established in the laboratory of Dr Darell Bigner and supplied by him for these studies (Bigner et al, 1988). Before the initiation of these experiments, the cell line was demonstrated to be negative for mycoplasma by hybridization with a radiolabelled RNA probe for mycoplasma ribosomal RNA. Exponentially growing D-247 MG cells were harvested with 0.25% trypsin–0.02% EDTA and

*Received 7 May 1997*
*Revised 18 August 1997*
*Accepted 22 August 1997*

*Correspondence to:* MR Zalutsky, Department of Radiology, Duke University Medical Centre, Durham, NC 27710, USA

viable cells were counted by trypan blue exclusion. RPMI-1640 medium (components purchased from JRH Biosciences, Lenexa, KS, USA) supplemented with 10% fetal calf serum, 2 mm L-glutamine, penicillin (50 U $ml^{-1}$) and streptomycin (50 μg $ml^{-1}$) was used for all experiments and hereafter is designated as complete medium. After determining their viability, $2 \times 10^6$ cells were added to 80 ml of complete medium in stirrer flasks at 30 r.p.m. and placed in an incubator at 37°C in a 5% carbon dioxide atmosphere. Spheroids were used in experiments 7 or 8 days after the start of the culture. Measurements of the surface area at the maximum diameter of each spheroid, made with the NIH image program, were used to calculate spheroid radius and volume. The spheroids used in the higher activity concentration experiment had an average radius of 103 ± 10 μm and an average volume of $(4.69 \pm 1.54) \times 10^6$ $\mu m^3$ at the start of the experiment.

### Radiolabelled MAb

These experiments used the human/mouse chimeric construct of 81C6 (ch81C6) consisting of the human $IgG_2$ constant regions and murine 81C6 variable regions. The construction, purification and characterization of ch81C6, which reacts with the extracellular matrix antigen tenascin, has been described in a previous publication (He et al, 1994). Astatine-211 was produced via the $^{209}Bi(\alpha,2n)^{211}At$ reaction at the Duke University Medical Center cyclotron by irradiating bismuth metal targets with 28-MeV α-particles (Larsen et al, 1996). The $^{211}At$ activity was isolated from the cyclotron target by dry distillation into chloroform. *N*-succinimidyl 3-[$^{211}At$]astatobenzoate was synthesized, purified and coupled to ch81C6 according to previously published procedures (Zalutsky et al, 1994). The specific activity of $^{211}At$-labelled ch81C6 ranged between 30 and 52 kBq $\mu g^{-1}$. Sodium [$^{125}I$]iodide was obtained from DuPont New England Nuclear (North Billerica, MA, USA) and analogous procedures were used to produce $^{125}I$-labelled ch81C6 for autoradiography. An isotype-matched control MAb, TPS3.2 (Dangl et al, 1988), as well as ch81C6 were labelled with $^{125}I$ for the binding studies using a modification of the Iodo-Gen method (Fraker and Speck, 1978). Specific binding in vitro to D-54 MG tenascin-positive glioma homogenate was greater than 80% and trichloroacetic acid precipitation demonstrated that at least 98% of the radioactivity was protein-bound for all labelled ch81C6 preparations.

### Spheroid autoradiography

Spheroids were incubated with either 1 or 10 μg $ml^{-1}$ of $^{125}I$-labelled ch81C6 for 1–48 h at either 37 or 42°C. Spheroids were washed three times with fresh medium to remove unbound MAb, collected and embedded in Tissue-Tek OCT embedding compound (Miles, Elkhart, IN, USA) for the preparation of cryosections. Cryosections (10 μm) were mounted on glass slides coated with poly-L-lysine (Sigma). After fixation in methanol–isopropyl alcohol–acetone (50:40:10), slides were dipped in NTB-3 autoradiography emulsion (Kodak) and dried in a slanted position for several hours in the dark. The slides were then exposed at 4°C for 1 week. Slides were developed with Dektol developer and fixer (Kodak) according to the manufacturer's instructions. A standard haematoxylin and eosin counterstain was applied.

### Binding of ch81C6 to D-247 MG spheroids

Aliquots of ten spheroids of similar diameter to those used in the cytotoxicity assays were individually selected on an inverted microscope and placed in Eppendorf tubes with Hepes-buffered complete medium. Because unlabelled ch81C6 was added to some groups in the cytotoxicity assay, a group of spheroids were incubated with unlabelled ch81C6 at an about 40-fold excess for at least 1 h before the selection of spheroids for the binding analysis. This was carried out to determine the effect of the excess unlabelled MAb on binding of the radiolabelled ch81C6. Non-specific uptake of radiolabelled MAb was also assessed. For analysis of the kinetics of MAb binding to spheroids, $^{125}I$-labelled ch81C6 or $^{125}I$-labelled TPS3.2 were added to a final concentration of 250 kBq $ml^{-1}$ and spheroids were placed on a rotator at 37 or 42°C. At selected time intervals (1, 2, 4, 8 and 24 h), spheroids were removed and the medium was aspirated and twice replaced with fresh medium to remove unbound MAb. Excess medium was aspirated, and the spheroids transferred to a clean tube for counting of bound radioactivity in a gamma-counter (1282 Compugamma, LKB Wallac, Türkü, Finland). All measurements were performed in triplicate.

To assess binding as a function of mAb activity concentration, aliquots of ten spheroids were incubated for 1 h at 37 or 42°C with 7.8–125 kBq $ml^{-1}$ of $^{125}I$-labelled MAb. The MAbs were labelled with $^{125}I$ at the same specific activity as the $^{211}At$-labelled MAb preparations used in the cytotoxicity assays to allow direct comparison between $^{125}I$- and $^{211}At$-labelled MAbs. Spheroids were washed, transferred to clean vials and bound activity counted in the gamma-counter. All samples were performed in triplicate. Counts per minute per spheroid were graphed as a function of activity concentration in the medium.

### *Spheroid growth inhibition assays*

In some groups, unlabelled ch81C6 was added to the spinner culture flask at protein concentrations ranging from 10 to 187.5 μg $ml^{-1}$ at least 1 h before the addition of $^{211}At$-labelled ch81C6. This was carried out to inhibit specific binding of the $^{211}At$-labelled ch81C6 and thereby increase its penetration within the spheroid. These groups of spheroids, designated as low specific activity controls, contained at least an approximately 40-fold higher protein concentration of unlabelled MAb compared with the highest activity concentration of $^{211}At$-labelled ch81C6; at lower activity concentrations of radiolabelled MAb, this resulted in up to a 1000-fold excess of cold ch81C6.

A preliminary experiment was performed to determine a heating protocol that would not have a cytotoxic effect on these spheroids. As shown in Figure 1, 1 h of heating at 42°C did not decrease the growth rate of the spheroids, whereas longer hyperthermia treatments inhibited spheroid regrowth. Samples (3 ml) of the spheroids suspended in complete medium were aliquoted into tubes, $^{211}At$-labelled ch81C6 was added to the desired activity concentration (0.125 kBq $ml^{-1}$ to 250 kBq $ml^{-1}$), and the volume adjusted to 4 ml. The low specific activity controls continued to have unlabelled ch81C6 in the medium containing $^{211}At$-labelled ch81C6. Tubes were placed in an incubator at 37°C or 42°C on a rotator for 1 h. Then, the spheroids were washed twice using gravity sedimentation, resuspended in complete medium and placed in a sterile 15 × 60 mm Petri dish to select individual spheroids for plating.

 © Cancer Research Campaign 1998

**Figure 1** Effect of hyperthermia on spheroid regrowth; 37°C (open symbols), 42°C (closed symbols) incubated for 30 min (●); 1 h (■); 2 h (▲); 4 h (▼)

Adapting the methodology of Gaze et al (1992), spheroids were individually chosen and placed on soft agar (1.56%) overlaid with complete medium, one spheroid per well, using the centre eight wells of 24-well plates. The outer wells were filled with distilled water to minimize loss of medium by evaporation. Using a 20-µl pipette, 24 spheroids per treatment group were selected. After plating, the surface area at the largest diameter of each spheroid was measured using the NIH Image software program (v. 1.52), a Macintosh IIci personal computer and a Leitz microscope (Orthoplan) with a digitizing camera (Sony Model XC-711) as described below. Spheroids were measured every other day for 3 weeks, or until a 40-fold increase in volume was seen. (At larger volumes, the spheroids began disintegrating.) Spheroids were fed an additional 200 µl of complete medium per well once a week.

### Data analysis

The surface area at the maximum diameter of the spheroid, measured using the NIH Image program (v. 1.52), was used to calculate the radius and volume of each spheroid assuming spherical geometry. The mean and standard deviation of the volume of each treatment group ($n$ = 21–24 spheroids per group) was plotted vs days after treatment. The mean value of the spheroid volumes was chosen rather than the median because no spheroids were cured with this treatment protocol. Owing to contamination, only 16 and 13 spheroids, respectively, were measured at days 25 and 27 in the 42°C-plus-250 kBq ml$^{-1}$ treatment group. Growth curves were graphed on semilog axes, and a second-order regression fit and 95% confidence interval was obtained using Sigma Plot (Jandel Scientific, San Rafael, CA, USA). The time to reach a 25-fold increase in volume was calculated, as well as the 95% confidence interval for that time point. Previously published studies (Gaze et al, 1992) used a tenfold increase in volume as the end point; however, because of the rapid growth rate of D-247 MG spheroids (tenfold increase in volume in approximately 4–5 days), little effect on growth rate was seen at that end point. A 25-fold increase in volume was selected because that end point lay on the linear portion of the spheroid growth curve and the small initial volume of the spheroids allowed this magnitude of growth to be achieved. A $P \leq 0.05$ (i.e. at the 95% level) was considered to be significant.

### RESULTS

The effect of hyperthermia on spheroid growth at 42°C is presented in Figure 1. Hyperthermia treatments for time periods longer than 1 h resulted in a significant delay in spheroid growth. With longer heating periods, heating also affected spheroid physical integrity. After a 2-h incubation at 42°C, spheroids shed numerous individual cells over several days. Spheroids heated for 4 h lost physical integrity, resulting in a large number of single cells and cell clumps spread on the agar. This partial disintegration was manifested over the initial 10 days by an increase in spheroid diameter as seen by the digitizing camera and imaging program; however, visual inspection of the spheroids under the microscope revealed that the apparent growth was due to cell shedding. Even the spheroids heated for 4 h at 42°C eventually demonstrated evidence of regrowth, but in many cases spherical conformation was lost. Heating for more than 1 h also caused an apparent increase in the size of many of the cells.

The binding kinetics of ch81C6 to spheroids were measured with $^{125}$I- instead of $^{211}$At-labelled ch81C6 to avoid cytotoxicity at higher doses and/or longer incubation times, and to improve count rate at the conclusion of a 24-h study because of the longer half life of $^{125}$I. Even at an activity concentration of 250 kBq ml$^{-1}$, binding of $^{125}$I-labelled ch81C6 to D-247 MG spheroids continued to increase over 24 h, suggesting that antigen saturation had not occurred. After a 1-h incubation at 250 kBq ml$^{-1}$, an activity concentration of 7.1 Bq per spheroid was reached. Binding of $^{125}$I-labelled ch81C6 did not vary with temperature over the first 2 h, after which at 42°C it declined to the level reached by non-specific TPS3.2 MAb consistent with the compromised spheroid integrity at longer heating periods noted above. This study confirmed that specific binding of ch81C6 occurred by 1 h. Binding to spheroids as a function of medium activity concentration after a 1-h incubation is depicted in Figure 2. The absolute percentage of medium activity bound to the spheroids was low. For example, with 250 and 125 kBq ml$^{-1}$ ch81C6, $2.8 \times 10^{-3}$% and $5.0 \times 10^{-3}$% of the activity in the medium, respectively, was bound per spheroid at 37°C.

The growth curves for D-247 MG spheroids incubated for 1 h with 4–250 kBq ml$^{-1}$ of $^{211}$At-labelled MAbs at 37 and 42°C, as well as those not exposed to labelled MAb, are presented in Figure 3. At 4 and 8 kBq ml$^{-1}$, no effect of $^{211}$At-labelled ch81C6 on the growth rate of D-247 MG spheroids was observed at either temperature. Incubation of spheroids with 125 and 250 kBq ml$^{-1}$ of $^{211}$At-labelled ch81C6 significantly delayed spheroid growth compared with controls. Table 1 summarizes the number of days required for spheroids to achieve a 25-fold increase in volume for controls and groups incubated with 67.5, 125, and 250 kBq ml$^{-1}$ of $^{211}$At-labelled ch81C6. Similar trends were seen using a 30-fold increase in volume end point; however, these end points generally did not occur on the linear portion of the growth curve and the 95% confidence intervals were very large. The initial volumes of the spheroids in these treatment groups were quite similar (Table 1). Correlation coefficients for the fit of the second-order regression line ranged between 0.964 and 1.000. There was no significant growth delay compared with 37°C controls for spheroid groups (a) incubated at 42°C; (b) preincubated with unlabelled ch81C6

**Figure 2** Binding of $^{125}$I-labelled ch81C6 to spheroids after a 1-h incubation at varying media activity concentrations. Incubation at either 37°C (open symbols) or 42°C (closed symbols); (●, ○) $^{125}$I-labelled ch81C6; (■) spheroids preincubated with 40-fold excess unlabelled ch81C6 before the addition of $^{125}$I-labelled ch81C6; (▲) $^{125}$I-labelled TPS3.2. Error bars indicate one standard deviation. All measurements made in triplicate

**Table 1** Effect of $^{211}$At-labelled ch81C6 and temperature on D-247MG spheroid growth

| $^{211}$At activity (kBq ml$^{-1}$) | Pretreatment radius (μm)[a] | Time to reach 25 × volume (days)[b] |
|---|---|---|
| 37°C incubation | | |
| 0 | 107 ± 11 | 8.7 (8.1–9.2) |
| 67.5 | 103 ± 13 | 9.9 (9.2–10.7) |
| 67.5 (LSA)[c] | 105 ± 10 | 9.0 (8.6–9.4) |
| 125 | 103 ± 9 | 10.9 (9.9–12.0)* |
| 125 (LSA) | 103 ± 14 | 8.6 (8.4–8.9) |
| 250 | 99 ± 11 | 14.8 (13.2–16.5)* |
| 250 (LSA) | 100 ± 9 | 8.7 (8.5–8.9) |
| 42°C incubation | | |
| 0 | 107 ± 11 | 8.7 (8.2–9.2) |
| 67.5 | 103 ± 8 | 9.2 (8.7–9.7) |
| 67.5 (LSA) | 102 ± 9 | 8.7 (8.4–9.0) |
| 125 | 97 ± 9 | 10.5 (9.6–11.4)* |
| 125 (LSA) | 103 ± 14 | 8.8 (8.6–9.0) |
| 250 | 101 ± 9 | 16.0 (14.3–17.9)* |
| 250 (LSA) | 104 ± 11 | 8.6 (8.4–8.7) |

[a]Mean ± s.d; [b]mean value with 95% confidence interval; [c]low specific activity control, i.e. preincubated with excess of unlabelled ch81C6; *significantly different ($P < 0.05$) than no activity control.

(low specific activity controls); and (c) groups treated with 67.5 kBq ml$^{-1}$. None of the low specific activity control groups differed significantly from the temperature control groups. Exposure to 125 and 250 kBq ml$^{-1}$ of $^{211}$At-labelled ch81C6 resulted in a significant growth delay when compared with their temperature and low specific activity control groups. However, at all activity concentrations, hyperthermia had no effect on the growth rate of the spheroids. Increasing the activity concentration from 125 to 250 kBq ml$^{-1}$ resulted in a significant increase in growth delay at both temperatures (Figure 3). Autoradiographs (Figures 4 and 5) did not indicate that even prolonged heating (4 h) of the spheroids at 42°C increased MAb penetration.

### DISCUSSION

Local hyperthermia can improve the absolute level of MAb taken up by tumours in vivo (Stickney et al, 1987; Cope et al, 1990; Hauck et al, 1997). Although the mechanism by which hyperthermia exerts this effect is not known, alterations in MAb diffusion rate within the tumour interstitium as well as changes in antigen–MAb binding kinetics, vascular permeability and tumour blood flow all may play a role. Hyperthermia can enhance the cytotoxicity of low LET and low dose rate irradiation through inhibition of repair of radiation-induced damage and suppression of cell proliferation (Armour et al, 1991; Wang et al, 1992). Thus, concurrent hyperthermia treatment could be useful not only for increasing the tumour uptake of MAbs labelled with β-emitting radionuclides but also for potentiating their cytotoxicity. Moreover, as investigated in the current study, hyperthermia might enhance the cytotoxicity of α-emitting endoradiotherapeutic agents by increasing the homogeneity of their tumour uptake.

Multicellular tumour spheroids offer a three-dimensional tumour model of micrometastatic disease (Walker et al, 1988; Langmuir et al, 1990; Mairs et al, 1991; Gaze et al, 1992) in which to investigate this possibility without the influence of variable vascular flow and permeability as well as elevated interstitial fluid pressure, all of which can influence the uptake of targeted therapeutic agents (Baxter and Jain, 1989).

There is disagreement concerning the penetration of MAbs into tumour spheroids. Both specific and non-specific MAbs have been reported to penetrate into the interior of spheroids within 6 to 11 h (McFadden and Kwok, 1988; Hjelstuen et al, 1996). In contrast, it has been reported that intact MAbs targeted to cell membrane antigens bind only to the outer 1–3 cell layers of the spheroid, whereas MAb fragments penetrate 8–10 cell layers (Mairs et al, 1991). Chen et al (1991) demonstrated that a MAb that binds to nuclear histones penetrates to the necrotic centre of spheroids, implying that the hindrance to homogeneous distribution is not inherently a consequence of the size of MAbs but rather a function of their binding to the first available antigen.

Spheroids are a valuable model for the evaluation of endoradiotherapeutics because they better represent the barriers to homogeneous distribution in micrometastatic nodules than monolayer cultures. In addition, because of their three-dimensional structure, spheroids are an excellent model for studying the effect of cross-fire on cell kill with radionuclides. A mathematical model of MAb diffusion through tumour tissue has demonstrated the importance of using a three-dimensional model for evaluating distribution issues (El-Karch et al, 1993). By calculating the effect of interstitial components on diffusion, it was concluded that diffusion within a tumour nodule would be decreased by 10- to 20-fold compared with diffusion through water. The effects of impedance due to extracellular structures on the delivery of macromolecules must be addressed to investigate distribution on a cellular level. For these reasons, the tumour spheroid model was selected to investigate further the potential use of labelled MAbs in combination with hyperthermia, in particular as it might apply to the treatment of micrometastatic disease with $^{211}$At-labelled MAbs.

© Cancer Research Campaign 1998

**Figure 3** Growth of spheroids incubated at 37°C (closed symbols) or 42°C (open symbols) for 1 h at varying concentrations of $^{211}$At-labelled ch81C6. Second-order regression curves are presented. Low specific activity controls and intermediate activity concentrations eliminated for clarity. **(A)** (●, ○) No labelled mAb (temperature controls); (■, □) 4 kBq ml$^{-1}$ $^{211}$At-labelled ch81C6; (▲, △) 8 kBq ml$^{-1}$ $^{211}$At-labelled ch81C6; **(B)** (●, ○) No labelled mAb; (■, □) 125 kBq ml$^{-1}$ $^{211}$At-labelled ch81C6; (▲, △) 250 kBq ml$^{-1}$ $^{211}$At-labelled ch81C6

**Figure 4** Autoradiograph of $^{125}$I-labelled ch81C6 distribution in a D-247 MG spheroid after a 4-h incubation at 37°C taken at 250 × magnification. Section counterstained with haematoxylin and eosin

**Figure 5** Autoradiograph of $^{125}$I-labelled ch81C6 distribution in a D-247 MG spheroid after a 4-h incubation at 42°C taken at 250 × magnification. Section counterstained with haematoxylin and eosin

Although several investigators have achieved greater penetration of small molecules into spheroids by performing incubations under hypothermic conditions (Cody et al, 1993; Wartenberg and Acker, 1995), these results probably are not germane to our attempt to use hyperthermia to achieve the same goal. In these studies, increased penetration of fluorescent dyes such as SNARF-1 into spheroids was accomplished by performing incubations at 8–10°C. However, this effect was not related to increased molecular diffusion; at room temperature, the dyes were being trapped in the outer spheroid layers because of degradation by esterases.

Concurrent administration of 1 h of hyperthermia at 42°C to the D-247 MG spheroids failed to enhance the cytotoxicity of $^{211}$At-labelled ch81C6. In interpreting these results, it is important to consider several of the mechanisms by which hyperthermia potentially might enhance the effectiveness of endoradiotherapy. Hyperthermia can enhance the cytotoxicity of low LET radiotherapy through the inhibition of repair of sublethal damage; however, this should not be a factor with high LET α-particles such as those emitted by $^{211}$At (Gerner and Leith, 1977). Hyperthermia can modulate cell membrane antigen expression (Wong et al, 1989; Davies and Lindmo, 1990). Because ch81C6 recognizes an extracellular matrix protein, tenascin, this may decrease the likelihood that hyperthermia modulated its expression; nonetheless, shedding might occur. Finally, hyperthermia might alter the kinetics of MAb–antigen interaction; however, we have demonstrated that the binding kinetics of 81C6 to tenascin are the same over a temperature range of 37–45°C (Hauck et al, 1996).

Of particular relevance to the current study is the potential effect of hyperthermia on MAb diffusion through the tumour interstitium. The Stokes–Einstein equation for calculating the diffusion coefficient, $D = RT/(N_A 6\pi\eta\alpha)$, indicates that $D$ is directly proportional to the temperature (Curry, 1984). In addition, hyperthermia could decrease the viscosity of the interstitial fluid in the tumour (represented by η in the above equation). Given the short range of α-particles, improving the diffusion of a $^{211}$At-labelled MAb

© Cancer Research Campaign 1998 

should increase its cytotoxicity in tumours with radii greater than the 55–70 μm range of $^{211}$At α-particles.

With $^{211}$At-ch81C6, binding to the spheroid surface would be expected to result in cell kill to a maximum depth of 70 μm. Given the 100-μm initial radius of these spheroids, cell killing at the centre of the spheroid would require diffusion of the MAb into the spheroid, a process that we had hoped would be facilitated by hyperthermia. Unfortunately, there was no evidence for improved MAb distribution within the spheroids by hyperthermia, either measured indirectly by growth delay or by direct visual inspection of spheroid autoradiographs.

The effect of hyperthermia on MAb diffusion is complicated by the 'binding site barrier' (van Osdol et al, 1991) with the consequence that high affinity MAbs bind to the first antigen encountered, thereby retarding its diffusion to the deeper layers of the tumour. This is consistent with observations of higher penetration of MAb into tumour spheroids with lower affinity MAbs or lower antigen concentrations (McFadden and Kwok, 1988; Langmuir et al, 1992*b*). Because ch81C6 has a high affinity for tenascin ($K_A$ approximately $1.4 \times 10^9$ M$^{-1}$) (Hauck et al, 1996), the binding site barrier may have impeded its penetration with the D-247 MG tenascin-positive spheroids. Hyperthermia did not exacerbate this barrier to penetration as increasing the incubation temperature from 37°C to 42°C does not have a significant effect on the association or disassociation rate constants, or the equilibrium constant for the binding of radioiodinated 81C6 to D-247 MG glioma cells in vitro (Hauck et al, 1996).

Autoradiographs of these glioma spheroids exhibited patterns consistent with a binding-site barrier as binding of ch81C6 was largely confined to the outer layers of the spheroids (Figures 4 and 5). Although autoradiographs of spheroids might have been used to directly measure alterations in the depth of MAb penetration from a 1-h hyperthermia treatment, measurements taken on a spheroid section that did not include the maximum diameter would overestimate depth of penetration. Moreover, as enhancing cytotoxicity was the practical goal of this study, we chose to use growth delay as the end point to assess the effect of hyperthermia on MAb distribution.

Experiments were performed using unlabelled MAb in an attempt to saturate outer layers of antigen and facilitate penetration of the labelled MAb into the spheroid. Intact MAb was selected as the blocking agent instead of monovalent Fab fragments for several reasons. The lower molecular weight and avidity of the Fab should facilitate its diffusion into inner spheroid regions; however, it is the outer regions of the spheroid in which blocking of labelled MAb binding is required. In addition, because the affinity of 81C6 Fab is more than tenfold lower than intact MAb (Hauck et al, 1996), Fab bound to the outer spheroid layers would be a poor competitor for subsequently administered labelled intact MAb. Essand and colleagues (1995*a*,*b*) have demonstrated that increased penetration of labelled MAb into spheroids could be accomplished by previous administration of cold MAb. In DU 145 spheroids of about 800 μm diameter, this strategy increased labelled MAb delivery by about a factor of six at a penetration depth of 200 μm.

In the current study, no evidence for improved penetration of labelled MAb was seen either in the autoradiographs or in the growth delay experiments when spheroids were preincubated with an excess of cold MAb. In fact, the addition of unlabelled MAb had a deleterious effect as this tactic decreased growth delay in the 125 and 250 kBq ml$^{-1}$ groups. This presumably reflects competition of cold MAb for tenascin binding sites in outer spheroid layers. It is not clear why we were not able to improve spheroid penetration by blocking outer-layer binding with cold MAb. The amount of cold MAb that we used was at least 40 times higher than labelled MAb; the molar ratios of cold–labelled E4 MAb was 20:1 in one study (Essand et al, 1995*a*) and not indicated in the other (Essand et al, 1995*b*). Differences between our conditions and those of Essand et al (1995*a*,*b*) that might be relevant include our use of smaller spheroids (200 μm v 800 μm) and the fact that the 81C6 MAb reacts with a highly expressed, extracellular matrix antigen, whereas E4 binds to a cell surface antigen.

In summary, this study has demonstrated that a 1 h, 42°C hyperthermia treatment does not significantly improve cytotoxicity of $^{211}$At-labelled ch81C6 MAb in glioma spheroids as measured by spheroid growth delay. Longer hyperthermia treatments that enhance MAb uptake in vivo, such as 4 h at 41.8°C (Hauck et al, 1997), may still have a positive effect on the distribution of MAbs and, possibly, their cytotoxicity in vivo; however, the deleterious effects of prolonged hyperthermia treatment itself for these human glioma spheroids prevented the testing of these conditions in this in vitro model. Nonetheless, the significantly enhanced MAb tumour uptake and tumour-to-normal-tissue ratios obtained with hyperthermia in xenograft models suggests there may be a place for hyperthermia/labelled MAb combination therapy in the clinical armamentarium for cancer treatment. However, further studies are needed to better define the critical mechanisms responsible for hyperthermic enhancement of mAb uptake and cytotoxicity, as well as the optimal radionuclide for combining hyperthermia with radioimmunotherapy.

### ACKNOWLEDGEMENTS

This work was supported by National Institutes of Health Grants CA 42324, NS 20023, and Department of Energy Grants DE-FG05-95ER62021 and DE-FG05-96ER62148.

### REFERENCES

Armour EP, Wang Z, Corry PM and Martinez A (1991) Sensitization of rat 9L gliosarcoma cells to low dose rate irradiation by long duration 41°C hyperthermia. *Cancer Res* **51**: 3088–3095

Baxter LT and Jain RK (1989) Transport of fluid and macromolecules in tumors 1. Role of interstitial pressure and convection. *Microvascular Res* **37**: 77–104

Bigner SH, Mark J, Burger PC, Mahaley MS Jr and Bullard DE (1988) Specific chromosomal abnormalities in malignant human gliomas. *Cancer Res* **88**: 405–411

Bigner DD, Brown M, Coleman RE, Friedman AH, McLendon RE, Bigner SH, Wikstrand CJ, Pegram CN, Kerby T and Zalutsky MR (1995) Phase I studies of treatment of malignant gliomas and neoplastic meningitis with $^{131}$I-radiolabeled monoclonal antibodies anti-tenascin 81C6 and anti-chondroitin proteoglycan sulfate mel-14 F(ab′)$_2$ – a preliminary report. *J Neuro-Oncol* **24**: 109–122

Chen F-M, Hansen EB, Taylor CR and Epstein AL (1991) Diffusion and binding of monoclonal antibody TNT-1 in multicellular tumor spheroids. *J Natl Cancer Inst* **83**: 200–204

Cody SH, Dubbin PN, Beischer AD, Duncan ND, Hill JS, Kaye AH and Williams DA (1993) Intracellular pH mapping with SNARF-1 and confocal microscopy. I: a quantitative technique for living tissues and isolated cells. *Micron* **24**: 573–580

Cope DA, Dewhirst MW, Friedman HS, Bigner DD and Zalutsky MR (1990) Enhanced delivery of a monoclonal antibody F(ab′)$_2$ fragment to subcutaneous human glioma xenografts using local hyperthermia. *Cancer Res* **50**: 1803–1809

Curry F-RE (1984) Mechanics and thermodynamics of transcapillary exchange. In *Handbook of Physiology*, section 2, *The Cardiovascular System: The Microcirculation*, vol IV. Renkin EM and Michel CC (eds.), American Physiological Society: Betheseda, ML 309–374

© Cancer Research Campaign 1998

Dangl JL, Wensel TG, Morrison SL, Stryer L, Herzenberg LA and Oi VT (1988) Segmental flexibility and complement fixation of genetically engineered chimeric human, rabbit and mouse antibodies. *EMBO J* **7**: 1989–1994

Davies CDL and Lindmo T (1990) Hyperthermia-induced shedding and masking of melanoma-associated antigen. *Int J Hyperthermia* **6**: 1053–1064

El-Kareh AW, Braunstein SL and Secomb TW (1993) Effect of cell arrangement and interstitial volume fraction on the diffusivity of monoclonal antibodies in tissue. *Biophys J* **64**: 1638–1646

Essand M, Grönvik C, Hartmann T and Carlsson J (1995*a*) Radioimmunotherapy of prostatic adenocarcinomas: effects of $^{131}$I-labelled E4 antibodies on cells at different depth in DU 145 spheroids. *Int J Cancer* **63**: 387–394

Essand M, Nilsson S and Carlsson J (1995*b*) Uptake modification of a prostate-reactive monoclonal antibody in prostatic carcinoma spheroids. *Antibody Immunocon Radiopharm* **8**: 179–198

Fraker PJ and Speck JC (1978) Protein and cell membrane iodinations with a sparingly soluble chloroamide, 1,3,4,6-tetrachloro-3α-6α-diphenylglycoluril. *Biochem Biophys Res Commun* **80**: 849–857

Gaze MN, Mairs RJ, Boyack SM, Wheldon TE and Barrett A (1992) $^{131}$I-meta-iodobenzylguanidine therapy in neuroblastoma spheroids of different sizes. *Br J Cancer* **66**: 1048–1052

Gerner EW and Leith J (1977) Interaction of hyperthermia with radiations of different linear energy transfer. *Int J Radiation Biol* **31**: 283–288

Gridley DS, Ewart KL, Cao JD and Stickney DR (1991) Hyperthermia enhances localization of $^{111}$In-labeled hapten to bifunctional antibody in human colon tumor xenografts. *Cancer Res* **51**: 1515–1520

Hauck ML, Dewhirst MW and Zalutsky MR (1996) The effects of clinically relevant hyperthermic temperatures on the kinetic binding parameters of a monoclonal antibody. *Nucl Med Biol* **23**: 551–557

Hauck ML, Dewhirst MW, Bigner DD and Zalutsky MR (1997) Local hyperthermia improves uptake of a chimeric monoclonal antibody in a subcutaneous xenograft model. *Clin Cancer Res* **3**: 63–70

He X, Archer GE, Wikstrand CJ, Morrison SL, Zalutsky MR, Bigner DD, Batra SK (1994) Generation and characterization of a mouse/human chimeric antibody directed against extracellular matrix protein tenascin. *J Neuroimmunol* **52**: 127–137

Hjelstuen MH, Rasch-Halvorsen K, Brekken C, Bruland Ø and Davies CDL (1996) Penetration and binding of monoclonal antibody in human osteosarcoma multicell spheroids. *Acta Oncol* **35**: 273–279

Humm JL and Cobb LM (1990) Nonuniformity of tumor dose in radioimmunotherapy. *J Nucl Med* **31**: 75–83

Langmuir VK, Atcher RW, Hines JJ and Brechbiel MW (1990) Iodine-125-NRLU-10 kinetic studies and bismuth-212-NRLU-10 toxicity in LS174T multicell spheroids. *J Nucl Med* **31**: 1527–1533

Langmuir VK, Mendonca HL, Vanderheyden J-L and Su FM (1992*a*) Comparisons of the efficacy of 186Re- and $^{131}$I-labeled antibody in multicell spheroids. *Intl J Radiat Oncol Biol Phys* **24**: 127–132

Langmuir VK, Mendonca HL and Woo DV (1992*b*) Comparisons between two monoclonal antibodies that bind to the same antigen but have differing affinities: uptake kinetics and $^{125}$I-antibody therapy efficacy in multicell spheroids. *Cancer Res* **52**: 4728–4734

Larsen RH, Wieland BW and Zalutsky MR (1996) Evaluation of an internal cyclotron target for the production of $^{211}$At via the $^{209}$Bi (α,2n)$^{211}$At reaction. *Appl Radiat Isotop* **47**: 135–143

McFadden R, Kwok CS (1988) Mathematical model of simultaneous diffusion and binding of antitumor antibodies in multicellular human tumor spheroids. *Cancer Res* **48**: 4032–4037

Mairs RJ, Angerson W, Gaze MN, Murray T, Babich JW, Reid R, McSharry C (1991) The distribution of alternative agents for targeted radiotherapy within human neuroblastoma spheroids. *Br J Cancer* **63**: 404–409

Stickney DR, Gridley DS, Kirk GA, Slater JM (1987) Enhancement of monoclonal antibody binding to melanoma with single dose radiation or hyperthermia. *Natl Cancer Inst Monographs* **3**: 47–52

van Osdol W, Fujimori K, Weinstein JN (1991) An analysis of monoclonal antibody distribution in microscopic tumor nodules: consequences of a 'binding site barrier'. *Cancer Res* **51**: 4776–4784

Walker KA, Murray T, Hilditch TE, Wheldon TE, Gregor A and Hann IM (1988). A tumour spheroid model for antibody-targeted therapy of micrometastases. *Br J Cancer* **58**: 13–16

Wang Z, Armour EP, Corry PM and Martinez A (1992) Elimination of dose-rate effects by mild hyperthermia. *Intl J Radiat Oncol Biol Phys* **24**: 965–973

Wartenberg M and Acker H (1995) Quantitative recording of vitality patterns in living multicellular spheroids by confocal microscopy. *Micron* **26**: 395–404

Wong JY, Mivechi NF, Paxton RJ, Williams LE, Beatty BG, Beatty JD and Shively JE (1989) The effects of hyperthermia on tumor carcinoembryonic antigen expression. *Intl J Radiat Onc Biol Phys* **17**: 803–808

Zalutsky MR (1994) Radionuclide therapy: a review. In *Hadrontherapy in Oncology*, Amaldi U, Larsson B (eds), pp. 664–676 Elsevier Science: Amsterdam

Zalutsky MR, McLendon RE, Garg PK, Archer GE, Schuster JM and Bigner DD (1994) Radioimmunotherapy of neoplastic meningitis in rats using an α-particle-emitting immunoconjugate. *Cancer Res* **54**: 4719–4725

© Cancer Research Campaign 1998